# Stress Corrosion Cracking of an Austenitic Stainless Steel in Nitrite-Containing Chloride Solutions

**DOI:** 10.3390/ma7127799

**Published:** 2014-12-05

**Authors:** R. K. Singh Raman, Wai Hoong Siew

**Affiliations:** 1 Department of Mechanical and Aerospace Engineering, Monash University, Melbourne, Victoria 3800, Australia; 2 Mechanical Engineer, IBM Australia, 60 City Road, Melbourne, Victoria 3006, Australia; E-Mail: whs6211@gmail.com

**Keywords:** chloride stress corrosion cracking, austenitic stainless steel

## Abstract

This article describes the susceptibility of 316L stainless steel to stress corrosion cracking (SCC) in a nitrite-containing chloride solution. Slow strain rate testing (SSRT) in 30 wt. % MgCl_2_ solution established SCC susceptibility, as evidenced by post-SSRT fractography. Addition of nitrite to the chloride solution, which is reported to have inhibitive influence on corrosion of stainless steels, was found to increase SCC susceptibility. The susceptibility was also found to increase with nitrite concentration. This behaviour is explained on the basis of the passivation and pitting characteristics of 316L steel in chloride solution.

## 1. Introduction

One of the most accepted mechanisms of stress Corrosion cracking (SCC) (*i.e.*, the “dissolution-repassivation” mechanism [1,2,3]) requires recurrence of the steps of: (a) the stress-assisted disruption of the passive film at the crack-tip; (b) localized crack-tip dissolution at a high rate; and (c) repassivation at the crack-tip. Pitting is the one of the common SCC-initiators whereas the localized crack-tip dissolution facilitates crack propagation. The development of a locally conducive electrochemistry can trigger and sustain pitting, whereas an intricate synergy of the local electrochemistry with an applied/residual tensile stress at the crack tip is essential for the corrosion-assisted propagation of cracks [4]. Therefore, both pitting and cracking can be extremely sensitive to variations in electrochemistry. For example, caustic crack propagation rate of a steel was reported [5,6] to be 25 times greater in a 3.5M caustic solution containing 0.4M sulphide than in the sulphide-free solution. However, the industrial practices for mitigation of pitting and assisted cracking are often developed on the basis of data generated in plain solutions, and minor but critical variations in solution chemistry are often ignored. Therefore, it is not surprising that the limited laboratory data on pitting/cracking susceptibility do not always corroborate the varied experience in processing plants.

The profound role of the environment chemistry in pitting/cracking susceptibility is relevant also for the incidents of the pitting and cracking of stripping columns for a polymer processing plant that were constructed from a few highly corrosion resistant alloys (viz., super duplex stainless steel, Incoloy 825 and 316L stainless steel). Most notably, pitting and cracking in this instance had initiated and/or accentuated particularly when a nitrite compound was used as an alternative chemical for arresting the polymerization reactions in the stripping step. Influence of addition of a few anion types (chromates, sulphates, molybdates and nitrates) to chloride solution on pitting and chloride cracking of stainless steels has been reported [7,8,9,10,11,12,13,14,15]. These investigations would suggest the addition of these anions to have an inhibiting effect in pitting of stainless steels.

A study by Newman and Ajjawi [8] on the influence of nitrate in growth of an artificial pit suggests that nitrate induces passivation under a salt film at certain potentials which depend on the nitrate-to-chloride ratio. In a subsequent study, Newman and Shahrabi [7] have suggested that the electro-reduction of nitrate anions may contribute to nitrogen enrichment on the active dissolution sites, improving pitting resistance. This model is consistent with the improvement in pitting resistance of nitrogen-bearing stainless steels. However, the considerable accentuation in pitting/cracking in the stripping column as a result of nitrite addition (described earlier) is not consistent with the reported inhibiting influence of nitrate. It may be pertinent also to refer to the authors’ recent work [16] on SCC of a duplex stainless steel (DSS) in 30% MgCl_2_ solutions at 180 °C, showing the nitrite addition to retard SCC susceptibility at 1400 and 2800 ppm but accelerate at 5600 ppm.

This paper presents results of slow strain rate testing of an austenitic stainless steel in chloride solutions with different levels of nitrite content.

## 2. Experimental

The test material (316L stainless steel) was received in rolled plate condition. Test samples were machined from the plates. The chemical composition (wt%) of 316L plates used in the present study is: C: 0.02, Cr: 16.6, Ni: 10.0, Mn: 1.69, Mo: 2.1, N: 0.04, Cu: 0.39, P: 0.03, Si: 0.39, Al: <0.001, S: 0.01, Fe: Balance. Microstructure of the duly polished steel surface was revealed by electrochemical etching (etchant: 10 wt. % oxalic acid, voltage: 20 V, current: 5 A, time: 100 s) and observed by optical microscopy. 

Slow strain rate testing (SSRT) was employed for determination of SCC susceptibility. With the use of a variable speed motor, the SSRT rigs provide different constant cross-head speeds as the motor speed is varied from 10% to 100% of the full motor speed, which corresponds to various strain rates in the range of 10^−7^ s^−1^.

SSRT specimens were machined perpendicular to the rolling direction of the plate. Uniaxial cylindrical tensile specimens had an outer diameter (OD) of 8.0 ± 0.2 mm, with a gauge section of 20.0 ± 0.2 mm length and 3.0 ± 0.2 mm OD. The gauge section was ground in the longitudinal direction progressively to a 1200 grit finish, rinsed with acetone/water and air dried. A layer of Teflon tape was applied on the specimens except for their gauge sections. However, special care was taken while applying the tape to ensure there was no crevice corrosion. After a final rinse with acetone, the specimen was immediately mounted into the corrosion vessel which was then filled with different test solutions at 150 °C. While 30 wt. % MgCl_2_ (~2.23 × 10^5^ ppm Cl^−^) constituted the primary test solution, some of the tests solutions were added with various concentrations of nitrite (in ppm by weight), in order to achieve different chloride-to-nitrite ratios, as described in Table 1. All solutions were prepared using laboratory grade chemicals and distilled water.

**Table 1:** Solutions used for electrochemical experiments to characterize the effects of nitrite anions.

Volume of 1 M NaNO_2_ added to 250 mL 30 wt. % MgCl_2_	Concentration of NO_2_^−^ anions (ppm)
0	0
10	1400
20	2800
40	5600

While immersed in the test solution and subjected to straining at a constant rate, tensile stress was measured using a load cell and recorded every 10 min by a data-taker. All tests were conducted under open circuit potential conditions and the tensile specimen insulated from the grips and autoclave body by using appropriate Teflon and ceramic insulators. Fractographic examinations of specimens after completion of SSRT tests were carried out using scanning electron microscopy (SEM) in order to investigate the fractographic features of SCC. 

For understanding the electrochemical influence of nitrite addition, a few anodic polarization runs were also carried out, using a typical three-electrode system and a Princeton Applied Research (PAR) 273 potentiostat. An external saturated calomel reference electrode (SCE) was used in conjunction with a fine-sinter glass luggin while a single graphite rod was used as the counter electrode. The working electrode consisted of machined cylindrical rods of approximately 30 mm in length and 8 mm outer diameter (OD). A wetted surface area of approximately 0.19 cm^2^ was defined as the area tested. Prior to installation in the corrosion cell, the working electrode end was wet ground with silicon carbide paper to a 1200 grit finish, rinsed with distilled water followed by a degreasing rinse with acetone and subsequently air-dried.The test solution consisted of 150 mL 0.1M NaCl mixed with varying volumes of 0.1M sodium nitrate (NaNO_3_). The desired temperature of the working solution was set and maintained for the duration of the experiment. All solutions were prepared from analytical grade reagents and distilled water, and were used in their naturally aerated state.Prior to every polarisation scan, a 30 s period of conditioning at −1.00 V was performed to remove the passive layer on the alloy surface. After conditioning, a delay of two hours allowed for open circuit potential (OCP) stabilisation. All scans were started at −250 mV (with respect to OCP) and continued onwards to the anodic region until the onset of transpassivity.

## 3. Results and Discussion

**Figure 1:**
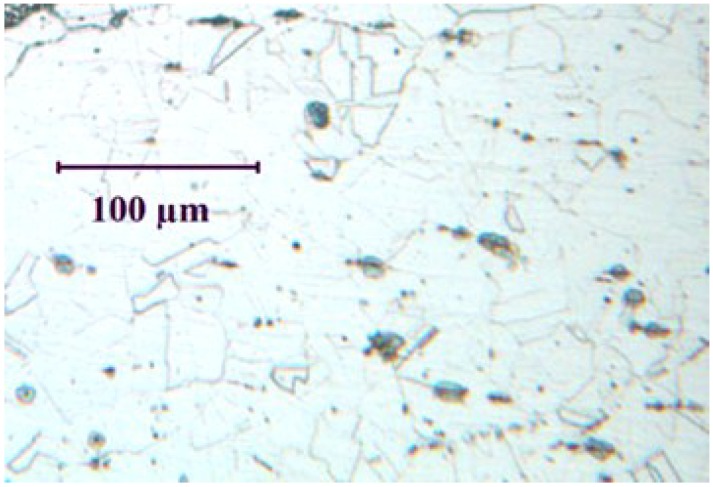
Etched microstructure of 316L stainless steel used in the present study.

The etched microstructure of the 316L stainless steel (shown in Figure 1) consists primarily of austenite grains. 

Two sets of slow strain rate testing (SSRT) experiments were carried out, the first set for identifying the strain rate or the range of strain rates that produces SCC in plain 30% MgCl_2_ solution, and the second set for comparison of SCC susceptibility in 30% MgCl_2_, with and without nitrite addition (at a strain rate as identified through the first set of experiments).

### 3.1. SSRT in Plain MgCl_2_ Solution at Various Strain Rates

**Figure 2:**
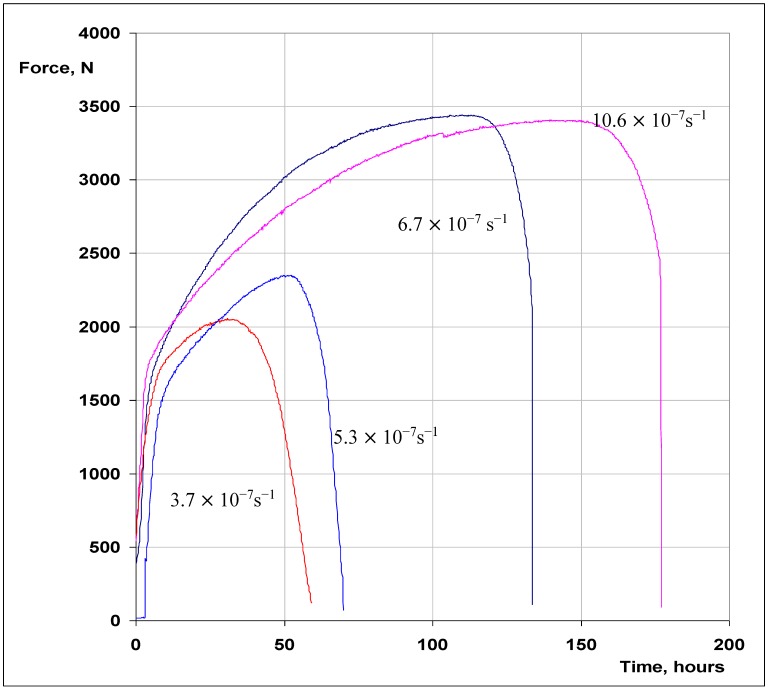
Force *vs.* time slow strain rate testing (SSRT) curves of 316L in 30 wt. % MgCl_2_ at 150 °C, at different strain rates.

**Figure 3:**
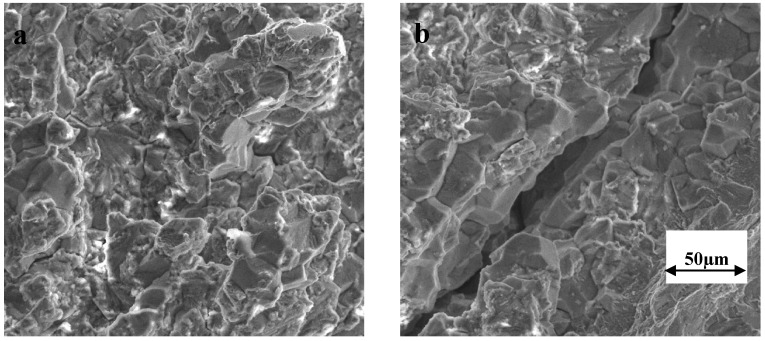
SEM fractograph of 316L tested in 30 wt. % MgCl_2_ at 150 °C, showing intergranularstress corrosion cracking (SCC) that covered a considerable fraction of the fracture surface, when tested at strain rates: (**a**) 3.7 × 10^−7^ s^−1^ and (**b**) 5.3 × 10^−7^ s^−1^.

**Figure 4:**
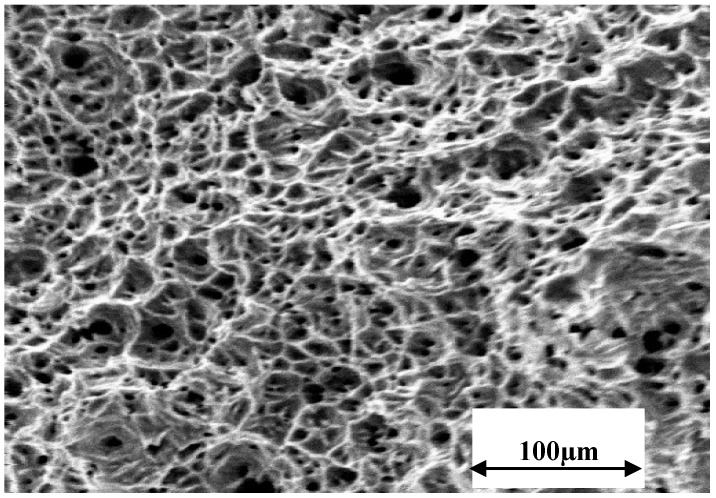
A representative SEM fractograph showing purely ductile failure.

Figure 2 presents the force *vs.* time curves for the SSRT specimens tested at 150 °C at 30%, 40%, 50% and 70% of the full motor speed, which respectively correspond to the strain rates of 3.7 × 10^−7^, 5.3 × 10^−7^, 6.7 × 10^−7^ and 10.6 × 10^−7^ s^−1^. It is evident from Figure 2 that time to failure (t_f_) is considerably lower at 3.7 × 10^−7^, 5.3 × 10^−7^, 6.7 × 10^−7^ s^−1^ in comparison to that at 10.6 × 10^−7^ s^−1^. While the shorter t_f_ at 10.6 × 10^−7^ s^−1^ may simply be attributed to the strain rate, the considerably longer t_f_ for the specimen tested at 6.7 × 10^−7^ s^−1^ in comparison to the specimen tested at 3.7 × 10^−7^ and 5.3 × 10^−7^ s^−1^ is a strong indication of SCC at 3.7 × 10^−7^ and 5.3 × 10^−7^ s^−1^. Indeed, the SEM fractography revealed the features of intergranular SCC (Figure 3) on a considerable fraction of the fracture surfaces of specimens tested at 3.7 × 10^−7^ and 5.3 × 10^−7^ s^−1^. The remainder of the fracture surfaces of these specimens had features indicative of only mechanical failure (*i.e.*, ductile dimples, as shown in Figure 4). On the other hand, the entire fracture surface of the specimens tested at 6.7 × 10^−7^ and 10.6 × 10^−7^ s^−1^ only had ductile dimples (similar to the features shown in Figure 4), *i.e.*, no areas with features of intergranular cracking were detected on these specimens. These results suggest the strain rates of 3.7 × 10^−7^ s^−1^ and 5.3 × 10^−7^ s^−1^ to be suitable for causing intergranular cracking to 316L immersed in 30% MgCl_2_ at 150 °C.

### 3.2. SSRT in Nitrite-Containing MgCl_2_ Solution

SSRT runs carried out using plain 30 wt. % MgCl_2_ solution at 150 °C have clearly resulted in intergranular SCC when strain rates of 3.7 × 10^−7^ s^−1^ and 5.3 × 10^−7^ s^−1^ were employed (as reported in Section 3.1). Accordingly, all SSRT tests for investigating the role of nitrite addition to the 30 wt. % MgCl_2_ solution were carried out only at 3.7 × 10^−7^ s^−1^.

**Figure 5:**
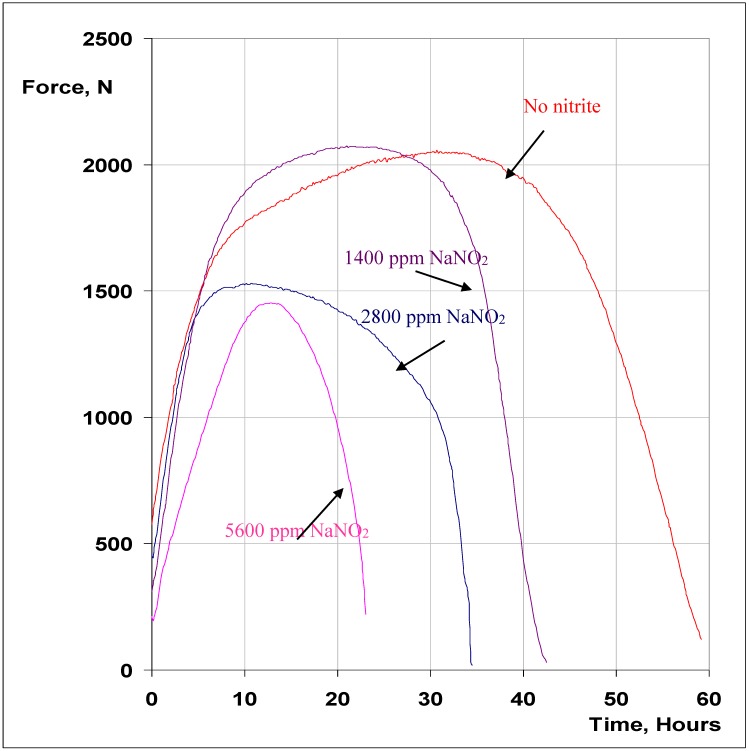
Force *vs.* Time SSRT curves (at strain rate of 3.7 × 10^−7^ s^−1^) for 316L in 30 wt. % MgCl_2_ at 150 °C, with different concentrations of NO_2_^−^.

**Figure 6:**
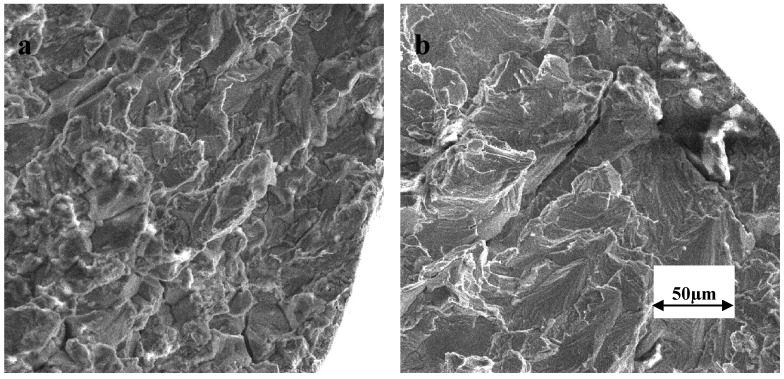
SEM fractograph of 316L tested in 30 wt. % MgCl_2_ at 150 °C at 3.7 × 10^−7^ s^−1^, showing: (**a**) intergranular SCC (IGSCC) in solution with 0, 1400 and 2800 ppm NO_2_^−^, and (**b**) trans-granular SCC (in addition to IGSCC) in solution with 5600 ppm NO_2_^−^.

Figure 5 presents the force *versus* time curves for SSRT specimens tested in the plain MgCl_2_ solution as well as in the MgCl_2_ solution with the addition of 1400, 2800 and 5600 ppm (by wt.) of NO_2_^−^ anions. Intergranular cracking (Figure 6a) and transgranular cracking (Figure 6b), as shown in the representative SEM fractographs confirm the presence of SCC in each case. The addition of NO_2_^−^ to the chloride solution clearly accelerates the SCC failure. The SSRT plots show a systematic increase in SCC-susceptibility with increase in NO_2_^−^ content, as indicated by the systematic increase in t_f_ with decreasing NO_2_^−^, *i.e.*, 5600 ppm > 2800 ppm > 1400 ppm (Figure 5). Maximum force sustained was also considerably lower in the case of specimens tested in solutions with 2800 and 5600 ppm NO_2_^−^. SEM fractography has also suggested that while the samples tested in solutions with 0, 1400 and 2800 ppm NO_2_^−^ produced only intergranular SCC (Figure 6a), the fractographs of the samples tested in solutions with 5600 ppm NO_2_^−^ also had significant areas of trans-granular SCC (in addition to intergranular SCC), as shown in Figure 6b.

The increase in SCC susceptibility with the nitrite addition (Figure 5) may be attributed to the ability of the nitrite contents used in this study to provide just enough passivation that may be necessary for sustaining the stress corrosion cracking. It may be rudimentary but still very relevant to recall here that a relatively less stable passive film would facilitate the sustenance of the SCC (for the systems susceptible to SCC by dissolution-repassivation mechanism).

**Figure 7:**
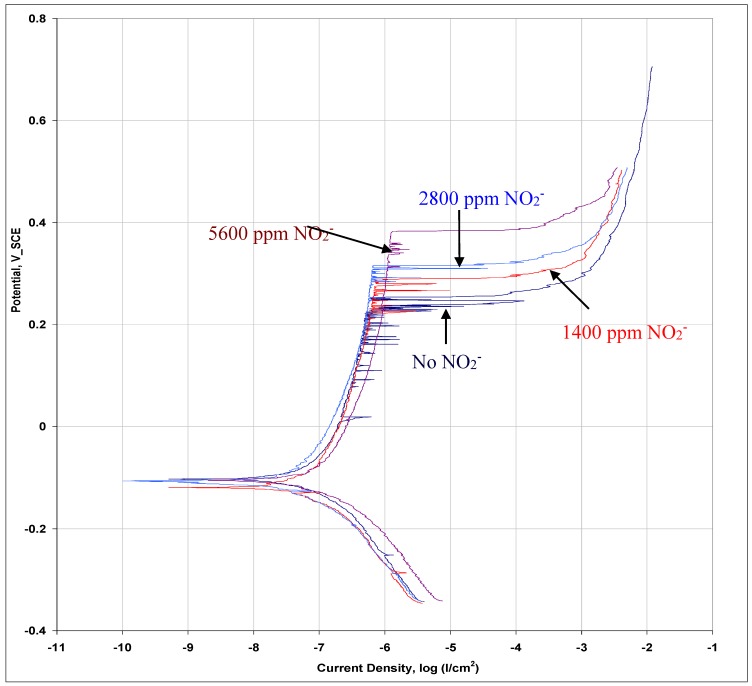
Anodic polarization curves of 316L in 0.1M NaCl with 0, 1400, 2800, and 5600 ppm NO_2_^−^.

This effect appears to increase with increasing nitrite content in the range of 1400–5600 ppm. A few anodic polarization tests using solutions with 1400, 2800 and 5600 ppm of NO_2_^−^ (Figure 7) suggest extensive meta-stable pitting activity in the solution with no NO_2_^−^, and a systematic decrease in the activity with increasing NO_2_^−^ content. Also, a systematically increasing positive shift in pitting potential was observed with the increase in the NO_2_^−^ content. There is very little meta-stable pitting activity in the case of solution with 5600 ppm of NO_2_^−^, which may indicate a significant role of the pitting-independent SCC mechanism at high NO_2_^−^ contents. With increasing passivation characteristic upon increase in NO_2_^−^ content of the solution, a condition may establish for cracks to initiate and propagate also within grains (*i.e.*, trans-granular cracking), *i.e.*, without the need of grain boundary pitting. Of the solutions used, this aspect will be facilitated most in the solution with 5600 ppm NO_2_^−^ and hence the considerable area of trans-granular cracking (Figure 6b). Thus the initiation of stress corrosion cracks does not need to rely on grain boundary pitting, which is consistent with the enhanced SCC with increasing NO_2_^−^ addition.

The authors’ own work [16] on SCC of a duplex stainless steel (DSS), SAF 2507 in 30% MgCl_2_ solutions at 180 °C has suggested the nitrite addition to retard SCC susceptibility at 1400 and 2800 ppm. This behaviour is possibly a result of the much superior passivation characteristics and greater resistance to SCC and pitting (as a result of its greater Cr content and the dual phase structure of DSS). The electrochemical tests reported in the same paper [16] have shown that with the addition of 1400 and 2800 ppm NO_2_^−^ a robust passive layer develops, which resists its own cracking as well as SCC. Accordingly, it is believed that a much higher NO_2_^−^ content may be required for developing such a robust layer in the case 316L SS.

## 4. Conclusions

As established by slow strain rate testing (SSRT) of 316L stainless steel at various strain rates in 30% MagCl_2_ solution at 150 °C, with and without various NO_2_^−^ additions, and by the post-SSRT fractography:

(a) 316L suffers intergranular stress corrosion cracking (SCC) at strain rates of 3.7 × 10^−7^ and 5.3 × 10^−7^ s^−1^, and no SCC at higher strain rates.
(b) Additions of NO_2_^−^ (1400–5600 ppm) accelerate susceptibility to chloride SCC.
(c) With increasing NO_2_^−^ content, SCC susceptibility increases in the order, 1400 ppm > 2800 ppm > 5600 ppm. This behaviour has been attributed to the increasing passivation characteristic with increasing NO_2_^−^ content.
